# Association between rapid force production by the plantar flexors and balance performance in elderly men and women

**DOI:** 10.1007/s11357-016-9949-3

**Published:** 2016-08-31

**Authors:** Ryoichi Ema, Megumi Saito, Shunsuke Ohki, Hirokazu Takayama, Yosuke Yamada, Ryota Akagi

**Affiliations:** 1 Graduate School of Engineering and Science, Shibaura Institute of Technology, 307 Fukasaku, Minuma-ku, Saitama-shi, Saitama, 337-8570 Japan; 2 Research Fellow of Japan Society for the Promotion of Science, 5-3-1 Kojimachi, Chiyoda-ku, Tokyo, 102-0083 Japan; 3 College of Systems Engineering and Science, Shibaura Institute of Technology, 307 Fukasaku, Minuma-ku, Saitama-shi, Saitama, 337-8570 Japan; 4 Department of Nutritional Science, National Institute of Health and Nutrition, 1-23-1 Toyama, Shinjuku-ku, Tokyo, 162-8636 Japan

**Keywords:** Rate of torque development, Maximal voluntary contraction, Triceps surae, Single-leg standing, Physical activity, Sex difference

## Abstract

Plantar flexion strength and balance ability are considered to be crucial for avoiding falls. However, no clear relationship has been established between these two factors in elderly population. This study aimed to examine the association between plantar flexion strength and balance performance in elderly men and women. Forty-three men and 35 women aged over 65 years performed isometric plantar flexion as fast and hard as possible. From the time-torque curve, the rate of torque development in time intervals of 30, 50, 100, 150, and 200 ms from the onset of contraction was determined and normalized to peak torque. In addition, the center of pressure displacement during single-leg standing was calculated and normalized to height. When the data were collapsed over sexes, the normalized rate of torque development was negatively correlated with the normalized center of pressure displacement, except for the time interval of 200 ms. By sex, regardless of the time interval, there was a negative correlation between the normalized rate of torque development and the normalized center of pressure displacement in the elderly men but not in the elderly women. No correlation was seen between the peak torque and normalized center of pressure displacement in either pooled or separated data. The findings suggest that the capability of rapid force production rather than maximal force production of the plantar flexion is important for balance ability in elderly men, but this capability may not be relevant in elderly women.

## Introduction

Prevention of falls is essential for elderly people because falls can result in devastating problems such as fractures and incapacitation. Major risk factors for falls include impairments of muscle strength and balance performance (Rubenstein 2006). Compared with elderly non-fallers, elderly fallers have less ankle joint strength, especially plantar flexion strength (Cattagni et al. 2014; LaRoche et al. 2010) and poorer balance performance (Cattagni et al. 2014). These findings suggest that the force-generating capacity of the plantar flexors contributes markedly to balance ability, reducing the risk of falls. Indeed, maximal voluntary contraction (MVC) force of the isometric plantar flexion was related to balance performance in elderly people (Spink et al. 2011). It takes more than 300 ms to reach MVC force from the onset of muscle contraction (Thorstensson et al. 1976), whereas rapid postural recovery in less time than this is required when a loss of balance occurs (Pijnappels et al. 2005). Therefore, explosive plantar flexion force such as rate of force development (RFD), which is defined as the slope of the time-force/torque curve (Aagaard et al. 2002), may be related more closely to balance performance than MVC force. The importance of explosive force on postural control has been documented in previous studies (e.g., Blazevich et al. 2008; Folland et al. 2014; Jenkins et al. 2014; Kobayashi et al. 2016). To the best of our knowledge, however, the relationship between explosive plantar flexion force and balance performance has not been substantiated by experimental data among elderly people.

The purpose of the present study was to examine the association between plantar flexion strength and balance performance in elderly men and women. Considering the different training-induced responses between MVC force and RFD (Tillin et al. 2011) and between plantar flexion strength and balance performance (Kobayashi et al. 2016), clarification of this issue will be of benefit in establishing effective exercise programs for the improvement of balance performance as well as plantar flexion strength among elderly people.

## Methods

### Subjects

Plantar flexion strength and balance performance were measured in 43 elderly men and 35 elderly women aged over 65 years. Their physical characteristics are shown in Table 1. Independent *t* tests demonstrated no significant difference in age (*P* = 0.079) between the elderly men and women, whereas the height (*P* < 0.001) and body mass (*P* < 0.001) were significantly higher in the elderly men than in the elderly women. They were functionally independent in daily life. Some of the subjects were recreationally active, engaging in activities such as walking, light calisthenics, yoga, mini golf, table tennis, softball, or tennis once or twice a week. Other participants were sedentary and did not exercise. This study was approved by the Ethics Committee of the Shibaura Institute of Technology. The subjects were informed of the purpose and potential risks of the study and provided written informed consent.

**Table 1:** Physical characteristics of participants

		Elderly men (*N* = 43)	Elderly women (*N* = 35)
Age	Years	73 ± 5	71 ± 3
Height	cm	164.6 ± 6.9	153.2 ± 6.0^a^
Body mass	kg	63.2 ± 8.2	52.6 ± 7.1^a^
Body mass index	kg/m^2^	23.3 ± 2.5	22.4 ± 2.6

Data are presented as mean ± standard deviation

^a^Significant difference between elderly men and women

### Data acquisition and analysis

#### Plantar flexion strength

The MVC strength of the plantar flexion was measured with a dynamometer equipped with a torque transducer (TD200, Kubota Corporation, Japan) (Kobayashi et al. 2016). The measurement was performed on the leg that was the one contralateral to that used for kicking a ball. The subject sat on the bench of the dynamometer (the hip, knee, and ankle joints = 80°, 0°, and 0°, respectively; anatomical position = 0°), with the knee and foot secured to the bench or footplate with non-elastic straps. The centers of rotation of the dynamometer and the ankle joint were visually adjusted. After several submaximal plantar flexion contractions as a warm-up, the subject was instructed to perform plantar flexion as fast and hard as possible and to keep plantar flexion for about 3 s. The trials were conducted three times with sufficient rest between the trials, and the torque signals were stored in a computer through an A/D converter operating at 1 kHz (PowerLab16/35, ADInstruments, Australia). The torque signal was low pass filtered at 15 Hz using a fourth-order zero phase lag Butterworth filter (Aagaard et al. 2002). The peak value of each torque signal was defined as MVC torque. Thereafter, the onset of plantar flexion was determined as the instant when plantar flexion torque exceeded the baseline by 2.5 % of MVC torque (Aagaard et al. 2002; Thompson et al. 2014). The rate of torque development (RTD) was defined as the slope of the filtered time-torque curve over time intervals of 0–30, 0–50, 0–100, 0–150, and 0–200 ms from the onset of plantar flexion (Unhjem et al. 2015). We evaluated RTD at several time intervals because the major physiological determinants of RFD (Andersen and Aagaard 2006; Folland et al. 2014) and some group differences of RFD (Tillin et al. 2010) depended on the time intervals. In order to exclude the effect of MVC torque on RTD (Andersen and Aagaard 2006), RTD was normalized to MVC torque (normalized RTD). This value indicates the ability to rapidly develop force without the subject’s peak force capacity (Blazevich et al. 2008). The trial in which highest peak value of RTD was observed was used for the subsequent analyses. The mean of coefficient of variation (CV) and intraclass correlation coefficient type 1.1 (ICC [1,1]) of the highest and second-highest values were 11.8 % and 0.927 for peak value of RTD and 4.2 % and 0.972 for MVC torque, respectively.

#### Balance performance

The balance performance test with single-leg standing was performed with the eyes open. It is considered as a good indicator of balance ability (Billot et al. 2010) and is widely used to determine the balance ability in elderly people (Orr et al. 2008). The subjects were requested to stand barefoot with one leg on the platform of a center of pressure (COP) tracking device (T.K.K.5810, Takei Scientific Instruments, Japan) for 30 s. The measurement leg was the same as for strength testing. During standing, they looked at a point in front of them on the wall 2 m away at eye level. They were asked to hold their arms at the sides of their body and to lift the opposite foot with the knee flexed at about 90°. Three trials were performed with sufficient rest between the trials. If the subjects could not keep the raised foot lifted or the supporting leg on the platform, the trial was ended. The COP signals were sampled at 20 Hz and stored on a personal computer. The data of the first 5 s from the beginning of the onset were excluded to prevent any effect of postural changes on the COP trajectory. Thereafter, the COP displacement was determined in the time periods corresponding to 5–30 s, and the unit length was calculated (COP_TOTAL_). The COP_TOTAL_ was normalized to height (normalized COP_TOTAL_, Cattagni et al. 2014). The minimum unit length among the trials in which each subject could keep standing for 30 s was used for further analysis. Because men and women can control their posture differently depending on the direction (Kim et al. 2010), the COP displacements in the anteroposterior (COP_AP_) and mediolateral (COP_ML_) directions were also determined and normalized to height. When a subject could not remain standing for 30 s, the trial in which the subject could stand longest was selected for the analyses, and the displacement was determined until the time 3 s before the end of the trial from 5 s after the onset. If a subject could not stand for 10 s, the data for that subject were excluded from the analyses.

#### Muscle thickness

The muscle thickness of the triceps surae was determined during quiet standing at 30 % of the lower leg length from the popliteal crease to the lateral malleolus (Miyatani et al. 2004) using B-mode ultrasonography (SSA-770 aplio80, Toshiba Medical Systems, Japan) with a 60-mm linear-array probe. The measurement was conducted two times, and means were used for further analyses. The mean of CV and ICCs (1,2) of the repeated measurements were 0.5 % and 0.997, respectively.

#### Physical activity

After the strength and balance performance measurements, the subjects were requested to perform their routine daily activities while wearing a tri-axial accelerometer (Actimarker EW4800, Panasonic Electric Works, Japan) for 8 weeks, and physical activity in daily life was monitored. The accuracy of measurement of physical activity using the accelerometer has been confirmed previously (Yamada et al. 2009). This evaluation was conducted because the magnitude of physical activity during daily life has been suggested to be related to RTD (Hannah et al. 2012) and balance performance (Abe et al. 2014). The number of days of monitor use was considered sufficient to acquire reliable data of physical activity (Togo et al. 2008). The device was attached during daily activity, except for the time being spent in the bath and sleeping. The mean amount of physical activity (min/day) was determined using the concept of metabolic equivalents (METs) at low (1.5–2.9 METs), moderate (3.0–5.9 METs), and vigorous intensities (>6.0 METs), respectively (Makizako et al. 2015). The number of steps per each day was also monitored. If the subject forgot to wear the device on that day (i.e., the number of steps was zero on 1 day), the corresponding day’s data were excluded from the analyses. We confirmed that no subjects suffered from diseases or injuries that result in difficulty of leading a normal life during the monitoring period.

### Statistical analysis

Data are presented as means ± standard deviations (SDs). Analyses were performed using SPSS version 22 (IBM, USA). The relationship between MVC torque or normalized RTD and normalized COP_TOTAL_ displacement was tested using Pearson’s product moment correlation coefficient. Two-way analysis of variance (ANOVA) (sex [men and women] × phase [30, 50, 100, 150, 200 ms]) with repeated measures was used on RTD. The differences of COP_TOTAL_ displacement, MVC torque, muscle thickness of the triceps surae, and number of steps between elderly men and women were tested using independent *t* tests. To investigate the effect of sex and direction (anteroposterior and mediolateral) on COP displacement, two-way ANOVA with repeated measures was used. Two-way ANOVA with repeated measures was also performed to test the effects of sex and intensity (light, moderate, vigorous) on the amount of physical activity. When a significant interaction was detected, subsequent ANOVAs with Bonferroni multiple comparisons were performed to determine whether the variables differed between elderly men and women. Cohen’s d and partial *η* ^2^ (*η*_p_^2^) were shown as effect sizes for independent *t* test and ANOVA, respectively. Statistical significance was set at *P* < 0.05.

## Results

Three elderly men could not maintain a single-leg standing for more than 10 s, so the data of those subjects were excluded from the following results.

### Relationships between strength and balance performance

**Fig. 1:**
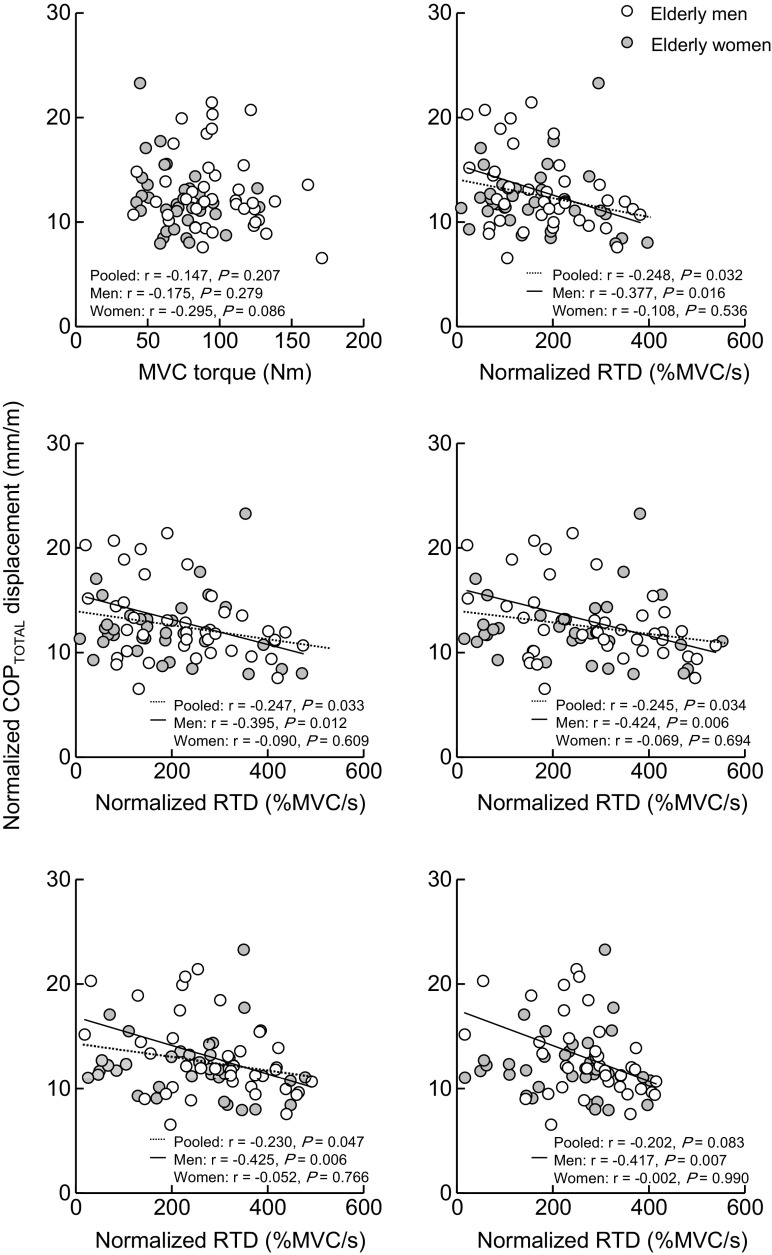
Relationship between MVC torque (*upper left*) or normalized RTD at time intervals of 30 (*upper right*), 50 (*middle left*), 100 (*middle right*), 150 (*lower left*), and 200 (*lower right*) ms from the onset of contraction and normalized COP_TOTAL_ displacement. *MVC* maximal voluntary contraction, *RTD* rate of torque development, *COP*_*TOTAL*_ center of pressure

Fig. 1 shows the relationship between MVC torque or normalized RTD and normalized COP_TOTAL_ displacement. No relationship was found between MVC torque and normalized COP_TOTAL_ displacement in either sex-pooled or -separated data. When the data were collapsed over sexes, there were significant relationships between normalized RTD and normalized COP_TOTAL_ displacement except for the time interval of 200 ms. In each sex, the corresponding relationships were significant in the elderly men at all phases but were not significant in the elderly women.

### RTD and MVC torque

**Fig. 2:**
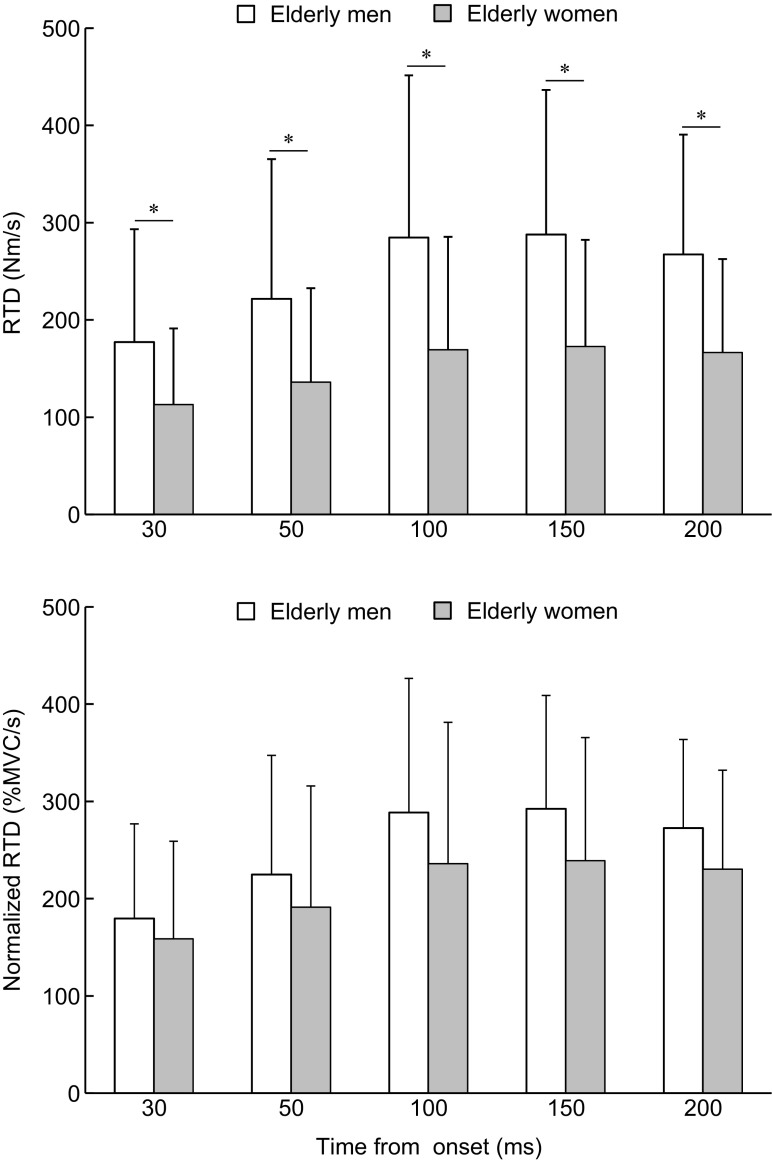
Rate of torque development (RTD) and RTD relative to maximal voluntary contraction (MVC) torque of the plantar flexion (normalized RTD). *Asterisk* indicates a significant difference between elderly men and elderly women. Data are presented as mean ± standard deviation

The RTD and normalized RTD are shown in Fig. 2. Two-way ANOVA showed significant main effects of sex (*P* = 0.001, *η*_p_^2^ = 0.145), phase (*P* < 0.001, *η*_p_^2^ = 0.467), and an interaction of the two factors (*P* < 0.001, *η*_p_^2^ = 0.071) on absolute RTD. The absolute RTD was significantly higher in the elderly men than women irrespective of phase (*P* <0.001–0.007, *η*_p_^2^ 0.095–0.173). On the other hand, there was no main effect of sex (*P* = 0.121, *η*_p_^2^ = 0.033) or interaction of sex × phase (*P* = 0.074, *η*_p_^2^ = 0.029) on normalized RTD. MVC torque was significantly higher (*P* < 0.001, Cohen’s d = 1.01) in the elderly men than women (Table 2).

**Table 2:** Strength, balance performance, and muscle size in elderly men and women

Measured variables		Elderly men	Elderly women
MVC torque	Nm	97.7 ± 29.0	72.0 ± 21.2^a^
COP_TOTAL_ displacement			
Absolute value	mm	21.1 ± 6.1	18.6 ± 4.5^a^
Normalized value	mm/m	12.9 ± 3.7	12.2 ± 3.1
COP_AP_ displacement			
Absolute value	mm	12.3 ± 4.6	10.2 ± 2.6
Normalized value	mm/m	7.5 ± 2.8	6.7 ± 1.8
COP_ML_ displacement			
Absolute value	mm	14.1 ± 3.5	13.3 ± 3.2
Normalized value	mm/m	8.6 ± 2.1	8.7 ± 2.2
Muscle thickness	mm	65.2 ± 4.9	60.5 ± 3.7^a^

Data are presented as mean ± standard deviation

*MVC* maximal voluntary contraction, *COP*_*TOTAL*_ center of pressure, *COP*_*AP*_ center of pressure in anteroposterior direction, *COP*_*ML*_ center of pressure in mediolateral direction

^a^Significant difference between elderly men and women

### Balance performance

The data of balance performance are presented in Table 2. Independent *t* tests revealed that COP_TOTAL_ displacement was significantly larger in the elderly men than women (*P* = 0.048, Cohen’s d = 0.47). In contrast, no sex difference was found in normalized COP_TOTAL_ displacement (*P* = 0.400, Cohen’s d = 0.20). Two-way ANOVA showed a significant interaction of sex × direction (*P* = 0.037, *η*_p_^2^ = 0.058) on normalized COP displacement but not on absolute COP displacement (*P* = 0.065, *η*_p_^2^ = 0.046) without a main effect of sex in either value (*P* 0.059–0.502, *η*_p_^2^ 0.006–0.048). Post-hoc analyses showed no significant difference in the normalized COP_AP_ or COP_ML_ displacements between sexes (*P* 0.156–0.789, *η*_p_^2^ 0.001–0.027).

### Muscle size

The data of the muscle thickness of the triceps surae are shown in Table 2. The muscle thickness of the triceps surae was significantly higher (*P* < 0.001, Cohen’s d = 1.08) in the elderly men than women.

### Physical activity

**Fig. 3:**
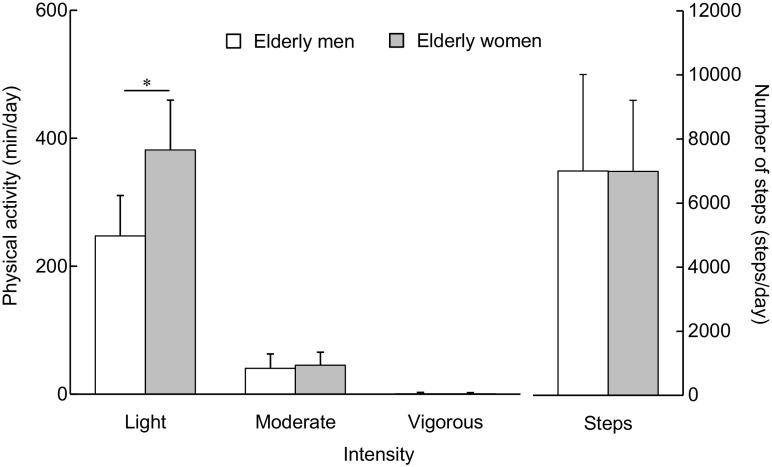
Amount of physical activity at light, moderate, and vigorous intensities and number of steps. The amount of physical activity was monitored for 8 weeks. *Asterisk* indicates a significant difference between elderly men and elderly women. Data are presented as mean ± standard deviation

The amount of physical activity is shown in Fig. 3. Two-way ANOVA demonstrated significant main effects of sex (*P* < 0.001, *η*_p_^2^ = 0.441), intensity (*P* < 0.001, *η*_p_^2^ = 0.947), and an interaction of the two factors (*P* < 0.001, *η*_p_^2^ = 0.474). The amount of physical activity at light intensity was significantly higher in the elderly women than men (*P* < 0.001, *η*_p_^2^ = 0.484). By contrast, there were no differences in physical activity at moderate (*P* = 0.348, *η*_p_^2^ = 0.012) or vigorous (*P* = 0.881, *η*_p_^2^ < 0.001) intensities. The number of steps did not differ significantly (*P* = 0.986, Cohen’s d < 0.001) between elderly men and women.

## Discussion

The present study found a correlation between normalized RTD and normalized COP_TOTAL_ displacement for elderly people. By sex, an association was observed for the elderly men, whereas no association was observed for the elderly women. The MVC torque was not correlated with normalized COP_TOTAL_ displacement in either sex-pooled or -separated data. These findings suggest that the capability of rapid force production rather than maximal force production of the plantar flexion is important for balance performance in elderly people, especially in elderly men, whereas the capability may not be relevant for elderly women.

The magnitude of physical activity may account for the lack of association between normalized RTD and normalized COP_TOTAL_ displacement in the elderly women (Fig. 1). In the current study, the magnitude of physical activity at light intensity was higher in the elderly women than men (Fig. 3), which is in line with a previous study (Gando et al. 2014). It was shown that low intensity training of the plantar flexion induced an increase in RTD but not in MVC torque (Gruber et al. 2007). Explosive plantar flexion training increased both MVC torque and RTD of the plantar flexion, whereas static balance performance with single-leg standing was not changed in the elderly population (Kobayashi et al. 2016). In addition, the magnitude of physical activity at vigorous, but not at light intensity, was associated with balance performance in middle-aged and elderly women (Abe et al. 2014). Based on these previous results, it is possible that the greater magnitude of physical activity at light intensity prevented age-related decline of RTD but was insufficient to preclude the impairment of MVC torque and balance performance in the elderly women. Indeed, additional simple regression analyses showed that age was negatively correlated with MVC torque (*r* = −0.386, *P* = 0.022) and normalized COP_TOTAL_ displacement (*r* = −0.584, *P* < 0.001) but not with normalized RTDs at any phases in the elderly women. Therefore, the effect of the physical activity on MVC torque, RTD, and balance performance may be different, affecting the associations between the parameters. Taken together, it is likely that the sex difference in the relationship between RTD and balance performance results from the sex difference in the magnitude of physical activity.

Another possible factor for the different relationship between normalized RTD and normalized COP_TOTAL_ displacement is the sex difference in postural sway during single-leg standing. In the current study, a significant interaction of sex × direction was shown on normalized COP displacement, indicating that the contribution of COP_ML_ displacement to the COP_TOTAL_ displacement was larger in the elderly women than in the elderly men. The result supports a previous observation that an age-related decrease in balance performance during quiet standing was notable in the mediolateral compared with anteroposterior directions in women (Kim et al. 2010). These different characteristics of postural control during single-leg standing between elderly men and women may be related to the lack of correlation between normalized RTD and normalized COP_TOTAL_ displacement in the elderly women.

Furthermore, the amount of variability of parameters affects the degree of correlation (Goodwin and Leech 2006); if the inter-individual variabilities of normalized RTD and normalized COP_TOTAL_ displacement were small in the elderly women, this may have led to the lack of correlation between the two parameters. The CV among the elderly women for normalized COP_TOTAL_ displacement (25.2 %) was slightly lower than that among the elderly men (28.9 %), which might result in the absence of a correlation in the elderly women. In contrast, with respect to normalized RTDs, the CVs were high in the elderly women (range, 44.2–65.3 %) compared with elderly men (range, 33.4–54.6 %) at all time intervals. Accordingly, it is difficult to explain the sex difference in the association between normalized RTD and normalized COP_TOTAL_ displacement in terms of the difference in magnitude of inter-individual variability of the parameters.

We showed that absolute RTD was greater in the elderly men than women (Fig. 2). Except for muscle size, we did not evaluate the neural and muscular determinants of RTD (Maffiuletti et al. 2016), but the sex difference of absolute RTD may be partly explained by the corresponding difference in the maximal plantar flexion strength which is related to the difference in the muscle size (muscle thickness) of the triceps surae. However, we found no sex differences in the normalized RTD irrespective of phase (Fig. 2), which is in line with a previous finding for young individuals (Hanna et al. 2012). We speculate that the greater magnitude of physical activity at light intensity in the elderly women might have obscured a sex difference in the normalized RTD. In contrast, there was a significant difference in absolute RTD between young and elderly men, whereas no corresponding difference was noted in normalized RTD (Jenkins et al. 2014; Thompson et al. 2013). Considering the current and previous studies, it is likely that the age-related decline of explosive force (Thelen et al. 1996) is due to an age-related reduction in maximal force, but age does not substantially affect the ability of rapid force generation in either men or women. Further studies are required to clarify the mechanisms of age-related decline of explosive force through comparisons with young populations of both sexes.

## Conclusion

The present study demonstrated that the rate of torque development relative to maximal voluntary contraction torque of the plantar flexion is associated with balance performance in elderly people, especially elderly men. On the other hand, no relationship was seen between maximal voluntary contraction torque and balance performance. The findings suggest that the capability of rapid force production of the plantar flexion rather than maximal force production is important for balance ability in elderly men, but the corresponding capability may not be significant in elderly women. Although a number of previous studies have mentioned the importance of explosive force generation for balance ability, little is known about the association between the two factors in elderly people. The current findings provide direct evidence supporting this notion and indicate that strength training targeted at increasing the ability to rapidly develop plantar flexion force can improve balance ability in elderly men, whereas different exercise regimens may be required to improve balance ability in elderly women.
